# The Hepatokine FGF21 Increases the Human Spermatozoa Motility

**DOI:** 10.3389/fendo.2022.775650

**Published:** 2022-02-24

**Authors:** Guillaume Bourdon, Anthony Estienne, Claire Chevaleyre, Christelle Ramé, Fabrice Guérif, Jean-Sébastien Brun, Claudine Vasseur, Gaelle Fromont, Ingrid Plotton, Diane Dufour-Rainfray, Erika Caldas-Silveira, Joëlle Dupont, Pascal Froment, Pierre-Henri Ducluzeau

**Affiliations:** ^1^INRAE, UMR 85 Physiologie de la Reproduction et des Comportements, Nouzilly, France; ^2^Service de Médecine et Biologie de la Reproduction, CHRU de Tours, Tours, France; ^3^Centre de fertilité, Pôle Santé Léonard de Vinci, Chambray-lès-Tours, France; ^4^Service d’Anatomie et Cytologie Pathologiques, CHRU de Tours, Tours, France; ^5^Molecular Endocrinology and Rare Diseases, University Hospital, Claude Bernard Lyon 1 University, Bron, France; ^6^Laboratoire de Médecine Nucléaire in vitro, CHRU de Tours, Tours, France; ^7^Unité d’endocrinologie-diabétologie-nutrition, CHRU de Tours, Tours, France

**Keywords:** human, spermatozoa, FGF21 (fibroblast growth factor 21), sperm moTility, metabolism diseases, fertility

## Abstract

Lifestyle, environment and excess body weight are not only associated with an increased risk of metabolic disorders, such as type 2 diabetes, but also to other pathological processes, such as infertility. A hormone produced mainly by the liver called fibroblast growth factor 21 (FGF21) is closely linked to the energy status and is increased in patients suffering from obesity or insulin resistance. Recently, FGF21 has been shown to be associated with female fertility disorders, but no or few data about the role of FGF21 on human male fertility has been described. In the present study, FGF21 was measured in the seminal fluid at a lower level in comparison to the blood level. Thus, in the present *in vitro* study, we aimed to decipher the FGF21 system in human semen. To evaluate the putative role of FGF21 on spermatozoa function, we incubated human spermatozoa with increasing concentrations of recombinant human FGF21. The FGF21 in seminal fluid is potentially produced by male reproductive tract tissues. In spermatozoa, the FGF21 signal was transduced by the two main receptors FGFR1-c and FGFR3 and the cofactor β-klotho, which are colocalized in the middle piece of spermatozoa and stimulated the PI3K/Akt and MAPK pathways. Finally, *in vitro* treatment by FGF21 significantly increased sperm motility and ATP levels. Concomitantly, exposure to FGF21 improved the oxidative stress, as a lower ROS level was observed. Overall, these results seem to indicate that the metabolic factor, FGF21, positively modifies the activity and quality of the parameters of human spermatozoa.

## Introduction

Infertility is a public health problem that affects about 15% of couples of childbearing age. In almost 50% of cases, the male factor is involved (1). Furthermore, public health authorities note an increase in the use of conception centers by patients with metabolic disorders, such as obesity/insulin resistance. Because of the current prevalence of obesity (up to 20% of adults), the question of improving the fertility rate score is of great interest. It is now well established that links exist between energy metabolism and reproductive activity, involving both nutritional and hormonal regulations. Changes in metabolic status result in alterations in hormonal signals (insulin, IGF-1, and hormones produced by adipocytes called adipokines) and nutrient flow (fatty acids, glucose, and amino acids), all acting directly or indirectly on the hypothalamic-pituitary-gonadal axis (2). Interestingly, recent data support that a hormone produced mainly by the liver called fibroblast growth factor 21 (FGF21), is involved in female fertility disorders such as polycystic ovary syndrome (3–6), suggesting its contribution to the control of female fertility and raises the question about its involvement in male fertility.

FGF21 is a metabolic hormone mainly produced by the liver and described in the 2000s. This peptide belongs to the fibroblast growth factor (FGF) family, which is constituted of 22 members divided into seven subfamilies depending on phylogeny and functions (7, 8). All FGFs have paracrine activity, except the FGF19/21/23 subfamily, which present the specificity to be endocrine factors in contrast to others FGFs. FGFs bind specific FGF receptors (FGFRs) associated with a cofactor. FGFRs include FGFR1, FGFR2, FGFR3 and FGFR4, and all these receptors participate in heparan sulfate (HS)-dependent signaling. However, only the FGF19/21/23 subfamily has been shown to require cofactors from the Klotho family (α-klotho and β-klotho) of transmembrane proteins. Thereby, FGF21 needs to bind to FGFR1c, FGFR3c or FGFR4 associated with the presence of the β-klotho (KLB) cofactor, conferring the specific FGF21 activity and cell signaling. In humans, FGF21 plasma levels are detected between 0.05 and 5 ng.ml^-1^ (8) and between 0.1 and 1 ng.ml^-1^ in mice (9). However, pronounced interindividual variations exist in both species. Thus, in patients with type II diabetes or obesity, plasma concentrations of FGF21 are significantly increased compared to control healthy subjects (10). It has also been shown that the expression of FGF21 is induced by the nuclear receptor, peroxisome proliferator-activated receptor α, known to be a major regulator of energy homeostasis (11). The binding of FGF21 to its receptor and the cofactor β-klotho, causes phosphorylation of ERK1/2 kinases, or leads to phosphorylation of AMPK. Through these pathways, FGF21 modulates the activity of several metabolic organs, including adipose tissue, the pancreas, muscle and brain. FGF21 is upregulated in both cases (lack or excess of energy) and regulates glucose and lipid homeostasis by promoting lipid catabolism, including lipolysis; fatty acid oxidation; and mitochondrial oxidative activity, resulting in the improvement of insulin sensitivity (12, 13). For example, in adipose tissue, after binding of FGF21 to its receptors, it induces an increase in metabolic protein such as SIRT1, PGC1-a as well as UCP1 ultimately leading to heat dissipation, a sign of lipolysis.

Several FGFs (FGF1, 2, 4, 5, 8 and 9) and all FGFRs have already been localized in the mouse testis, including Sertoli, Leydig, and germ cells (14–16), and also in human testis (17). The paracrine and local functions of these FGFs have been demonstrated during testis development to stimulate proliferation, survival or to contribute to the formation of the interstitial compartment of the testis (16, 18). However, published data are scarce regarding the endocrine FGF19/21/23 subfamily. A mouse model overexpressing FGF21 in the liver has shown a strong decrease in pituitary LH levels, leading to a delay in puberty associated with female infertility. In addition, the deletion of the *fgf21* gene in mice led to elevated levels of apoptotic germ cells in the testis, which could be rescued after administration of recombinant FGF21, leading to sperm production (19). These recent data also support a regulatory role of the FGF21 hormone on testis and male fertility. However, no data about the role of FGF21 on human semen have been described.

The current work aimed to evaluate the presence of FGF21 in semen samples raising the question about the role of FGF21 on the sperm function. We aimed to decipher the FGF21 system in human semen, by determining the localization of FGF21 receptors and their activities in human sperm cells. To evaluate the role of FGF21 on spermatozoa function, we incubated human spermatozoa with increasing concentrations of recombinant human FGF21.

## Materials and Methods

### Patients

Human blood and semen are issued from the following cohort “Fertiprotect”, including healthy men and their respective normal semen quality, according to the WHO (2010) guidelines. Exclusion criteria include seropositivity for HIV, HBV or HCV, smoking and male explained infertility (chemotherapy, varicocele or genital surgery). Patients were enrolled into the Assisted Reproductive Centers (Tours, FERTIPROTECT protocol) for couple infertility exploration during a medical consultation. Forty participants gave full-informed written consent to participate in the study, and ethical approval was obtained from the Ethics Committee of the Vinci Clinic and CHRU Bretonneau. Patients (n = 40, 29–53 years old) (**Supplementary Data Sheet 1**) were separated in two groups depending on the body mass index (BMI), with 18.5-25 kg/m^2^ considered as normal BMI (and noted BMI ≤ 25 kg/m^2^) and BMI ≥ 30 kg/m^2^ considered as obese (**Table 1**). However, blood and seminal fluid were recovered from the same individual in a group of 20 patients (BMI ≤ 25, n=10; BMI ≥ 30, n=10). Blood and seminal fluid analyses were obtained in fasted patients, and semen collection was obtained after a recommendation of 2-5 days of abstinence. For *in vitro* analysis, recombinant human FGF21 from Sigma-Aldrich was prepared in water (100 µg/mL, stock solution). The selective FGFR1 and FGFR3 inhibitor PD173074 was obtained from Tocris Bioscience (Minneapolis, MN, USA) and used at the concentration of 100 nM (20, 21). Analysis of FGF21 was performed on fresh washed human spermatozoa from others patients. Spermatozoa were exposed for 15 min (for Western blot analysis) or 30 min to 0–10 ng/mL recombinant human FGF21, as described in the legends.

**Table 1:** Biological and semen parameters of samples from the BMI ≤ 25 kg/m^2^ group and the BMI ≥ 30 kg/m^2^ group.

	Patients with BMI<25 (n=12)	Patients with BMI>30 (n=12)	Significance
			
			
**Age (years)**	34.91 ± 1.28 (29 - 43)	37.08 ± 1.83 (30 - 53)	**NS**
**BMI (kg/m2)**	23.97 ± 0.50 (22 - 25)	34.96 ± 1.36 (30 - 44)	**** p<0.0001
			
**fasting glucose (mmol/L)**	5.55 ± 0.06 (5 - 6)	5.27 ± 0.28 (5 - 6)	**NS**
**cholesterol (mmol/L)**	5.40 ± 0.54 (5 - 7)	4.57 ± 0.69 (3 - 6)	**NS**
**triglyceride (mmol/L)**	1.47 ± 0.80 (0 - 4)	1.72 ± 0.30 (1 - 2)	**NS**
			
**FSH (UI/L)**	4.32 ± 0.92 (3 - 8)	3.88 ± 0.87 (2 - 8)	**NS**
**TSH (mUI/L)**	1.92 ± 0.36 (1 - 3)	2.46 ± 0.61 (2 - 4)	**NS**
**LH (UI/L)**	4.58 ± 0.41 (4 - 6)	5.30 ± 0.93 (3 - 9)	**NS**
**prolactin (mUI/L)**	185.56 ± 71.18 (11 - 331)	109.24 ± 97.22 (4 - 498)	**NS**
**testosterone (nmol/L)**	20.43 ± 1.96 (15 - 29)	13.50 ± 1.28 (10 - 18)	**p<0.01
**estradiol (pmol/L)**	72.00 ± 8.08 (62 - 88)	121.00 ± 36.87 (77 - 231)	**NS**
			
**semen volume (mL)**	4.80 ± 0.48 (3 - 7)	3.42 ± 0.46 (2 - 8)	*p<0.05
**semen concentration (million/mL)**	39.78 ± 9.06 (11 - 95)	68.57 ± 15.75 (2 - 200)	**NS**
vitality (after 1h)	69.20 ± 5.42 (31 - 88)	70.85 ± 2.98 (45 - 84)	**NS**
**motility (rapid progression) (after 1h)**	35.00 ± 4.09 (20 - 57)	42.00 ± 3.84 (15 - 66)	**NS**

Data are expressed as mean ± SEM (range). NS, non-significant; *, p < 0.05; **, p < 0.01; ****, p < 0.0001.

### Hormone and Metabolites Assay

ATP concentrations was measured by using the CellTiter-Glo™ ATP Assay Kit (Promega, France), and total-cholesterol concentration was measured by using the spectrophotometric assays (Biolabo, France), according to the manufacturer’s instructions. Plasma and seminal fluid levels of FGF21 were measured using the commercial Human FGF-21 Quantikine ELISA Kit (Bio-Techne, France).

### Western Immunoblotting and Immunoprecipitation

Pellets of human spermatozoa were lysed [Tris 1 M (pH 7.4), NaCl 0.15 M, EDTA 1.3 mM, EGTA 1 mM, VO43−23 mM, NaF 0.1 M, NH2PO41%, Triton 0.5%] and the protein concentration of samples was measured using a kit bicinchoninic acid (BCA) protein assay (Interchim, Montluçon, France) and equal protein concentrations were electrophoresed (40µg). Saturation of membrane was done with Tris-Buffered Saline Tween buffer (0.05% of Tween 20 and 5% of milk) for 30 min at room temperature. Then, the membranes were incubated at 4°C overnight with the following antibodies (all diluted at 1/1000): phospho-Akt (ser473), Akt, phospho-ERK (Thr202/Tyr204), and ERK (Cell Signalling Technologies, USA). Experiments were performed on five different patients.

Immunoprecipitation of FGF21 in seminal fluid was performed on a pool of human seminal fluid devoid of spermatozoa (2 mL), which was incubated overnight with rabbit polyclonal anti‐FGF21 antibody (Thermo-Fisher Scientific, USA) and immunoprecipitated by 100 mg protein G agarose beads. After several washes, the immunoprecipitated proteins and depleted seminal fluid extract were analyzed by Western blot. Experiments were performed on five pools of patients. Detection of proteins was done by using chemiluminescence (Western Lightning Plus-ECL, Perkin Elmer, Villebon-sur-Yvette, France) with a G-box SynGene (Ozyme, St Quentin en Yvelines, France).

### Immunohistochemistry

Paraffin-embedded testis, epididymis, prostate and seminal vesicle samples were retrieved from autopsy specimens from the Department of Histopathology and Urology/Andrology of the CHRU Bretonneau Tours Hospital and Hospices Civils de Lyon, France, following approval by the ethical committees of these institutions. Sections (7 µm) of the following human tissues (testis, epididymis, prostate and seminal vesicle) were deparaffinized and rehydrated in xylene and in various baths containing decreasing concentrations of alcohol (100, 90, 75%) for 10 minutes for each step. Immunohistochemical slides were washed in a PBS bath and microwaved for 2–3 min in antigen unmasking solution (Vector Laboratories, Inc., AbCys, Paris, France). An incubation with PBS 1X/0.1 M Glycine for 15 minutes at room temperature, has been performed to ensure saturation of aldehyde groups. To permeabilize cells on sections, an incubation for 15 min with a solution of 0.1% Triton X-100 (w/v) in PBS has been done. Finally, all nonspecific binding sites have been obstructed in 2% BSA solution for 15 min. For FGF21 immunostaining, sections were incubated overnight at 4°C with PBS/1% bovine serum albumin (BSA) containing primary antibody against FGF21 (Sigma-Aldrich, USA) at a 1:100 final dilution. Then, the sections were incubated with a “ready to use” labelled polymer-HRP anti-rabbit for 30 min (DAKO Cytomation Envision Plus HRP System, Dako, Ely, UK). Visualization was achieved by incubation in a DAB peroxidase substrate solution (Invitrogen, Cergy-Pontoise, USA).

Fresh human spermatozoa were fixed in 4% paraformaldehyde (PFA)/PBS for 15 min, then washed in a PBS bath. Spermatozoa were permeabilized with PBS-Triton 0.1%, and nonspecific binding sites were blocked in 2% BSA for 15 min, then incubated for 60 min at room temperature with FGFR3 (Thermo-Fisher, USA), FGFR1, FGFR4 and FGF21 (Sigma-Aldrich, USA) and β-klotho (Thermo-Fisher, USA) antibodies at a 1:100 final dilution. Rabbit or mouse IgG (Sigma-Aldrich, USA) antibodies (Sigma-Aldrich, USA) were used as negative controls. Analyses were performed in five different patients.

### Computer-Assisted Semen Analysis

Before FGF21 incubation, fresh semen samples were washed and centrifuged at 1200 rpm), resuspended in DMEM (Ref D6546, Sigma-Aldrich, USA) (4500 mg/L glucose, sodium pyruvate, and sodium bicarbonate, without L-glutamine, without albumin and with 5% serum) and counted. From each patient, 5 million spermatozoa were incubated at 37°C in a water bath with increasing concentrations of recombinant FGF21 (0, 0.01, 0.1, 1, 10, and 100 ng/mL) for 30 min, with or without preincubation with the specific FGFR inhibitor PD173074 for 15 min. Sperm motility, as a percentage of motile spermatozoa, was evaluated by using a computer-assisted semen analyzer (CASA) (Hamilton-Thorne Sperm Analyser IVOS version 12.2l, Hamilton Thorne Biosciences, USA) with a Makler Counting Chamber (0.01 sq.mm/0.01 mm Deep). Three microscopic fields were analyzed, and a minimum of 200 spermatozoa per field were evaluated. The following parameters were measured: percentage of motile sperm, percentage of progressively motile spermatozoa, average path velocity (VAP, average velocity/smoothed average position of the spermatozoa), progressive velocity (VSL, straight line distance between the beginning and the end of the track), curvilinear line velocity (VCL, average velocity measured over the actual point-to-point track followed by the cell), straightness (STR, a measure of side-to-side movement of the VCL determined by the ratio VSL/VAP × 100), linearity (LIN, a measure of the departure of the cell track from a straight line), amplitude of lateral head (µm) (ALH), and beat cross frequency (BCF) (Hz). All results are presented in **Table 2**.

**Table 2:** Kinematic parameters of human spermatozoa exposed to FGF21.

		0 ng/mL rFGF21		0.01 ng/mL rFGF21		0.1 ng/mL rFGF21		1 ng/mL rFGF21		10 ng/mL rFGF21		PD173074		PD173074 + 10 ng/mL rFGF21	
VSL (µm/sec)	35.5 ± 2.1			27.1 ± 0.7		43.1 ± 2.9		43.6 ± 2.9		45.41 ± 4.0*		37.9 ± 1.6		36.0 ± 2.6	
VCL (µm/sec)	62.2 ± 2.6			71.6 ± 1.4		78.14 ± 2.6**		73.98 ± 2.3*		73.8 ± 4.6*		68.1 ± 2.0		67.9 ± 3.9	
VAP (µm/sec)	40.8 ± 1.9			37.1 ± 0.8		51.5 ± 2.3*		51.1 ± 2.9*		56.0 ± 6*		46.4 ± 2.0		44.1 ± 2.7	
ALH (µm)	2.8 ± 0.3			3.0 ± 0.1		3.2 ± 0.2		3.0 ± 0.1		3.0 ± 0.2		2.6 ± 0.1		3.0 ± 0.2	
LIN (%)	56.4 ± 2.5			54.9 ± 0.7		56.5 ± 3.0		59.4 ± 2.4		60.1 ± 1.5		59.3 ± 1.6		55.8 ± 1.8	
BCF (beats/sec)	20.3 ± 1.4			16.3 ± 0.4		19.7 ± 1.2		15.5 ± 1.1		17.2 ± 0.8		18.6 ± 1.2		18.7 ± 1.3	
STR (%)	84.3 ± 1.2			82.5 ± 0.8		83.0 ± 2.0		84.2 ± 1.2		83.6 ± 1.1		83.8 ± 0.9		83.6 ± 1.7	

Computer-assisted sperm analysis (CASA) of spermatozoa were performed after 30 min of recombinant human FGF21 exposition with or without preincubation with the selective FGFR1-3 inhibitor PD173074 for 15 min. The following kinematic parameters were measured : VSL, Straight-Line Velocity; VCL, Curvilinear Velocity; VAP, Average Path Velocity; ALH, Amplitude of Lateral Head; LIN, Linearity; BCF, Beat Cross Frequency; STR: VAP, Straightness. All results are expressed as Mean ± SEM, n=9. *p < 0.05, **p < 0.01, compared with 0 ng/mL rFGF21.

### Viability and Mitotracker Analysis Using Flow Cytometry

Sperm membrane integrity was assessed with dual fluorescent probes, SYBR-14 and propidium iodide (PI) (Live/Dead Sperm Viability Kit, InvitrogenTM, Eugene, OR, USA) and semen were analyzed by using flow cytometry (MoFlo Astrios^EQ^, USA). Mitochondrial activity was determined using a 200 nM mitotracker (Orange CM-H2TMRos, Invitrogen, Fisher Scientific, France) and samples were analyzed by using flow cytometry. Twenty thousand events were collected per sample. Only sperm emitting orange fluorescence (R1, R2) were classified with a high mitochondrial membrane potential (HMMP), which is associated with mitochondrial activity. Orange fluorescence is in y axis and SSC in x axis. We have separated a R1 population with a very high orange fluorescence and a mild fluorescence population R2, from the negative control (without mitotracker).

All results are presented in **Supplementary Data Sheet 4**.

### Measurement of ROS [Hydrogen Peroxide (H_2_O_2_)]

The contents of the ROS hydrogen peroxide (H2O2) in human sperm (n=4 patients per condition) was measured by Ros-Glo H2O2 assay kit (Promega, Charbonnières-les-Bains, France). Two million spermatozoa previously stimulated by FGF21 were incubated with H2O2 substrate solution during 4 hours. H2O2 present in samples degradate the H2O2 substrate into Luciferin Precursor and produce luminescence which is measured with a luminometer Luminoskan Ascent (Thermo-Fisher, USA). Luminescence is correlated with the concentration of H2O2 and the ROS activity as detailed in the Ros-Glo H2O2 assay kit.

### Intracellular Calcium Measurements

A total of 2 million spermatozoa were loaded in a 96 microwell plate for fluorescent plate reader analysis, and the kinetics of intracellular calcium measurement were performed after a 2 μM Fluo-4 AM incubation. Fluorescence was measured every 30 s. The intracellular calcium intensity was plotted as the percentage change in fluorescence (ΔF/F0, %) compared with baseline (F0).

### Acrosome Integrity

After a 30 minutes exposition with/without FGF21, spermatozoa were incubated with 10 μM calcium ionophore A23187 to induce acrosome reaction and stained with 25 μg/mL FITC-conjugated pisum sativum agglutinin (FITC-PSA; Sigma-Aldrich) for another 30 min at room temperature. The same experiment has been performed without calcium ionophore A23187. The percentage of acrosome reaction was estimated by counting 200 spermatozoa per patient. Only spermatozoa without FITC-PSA staining or FITC-PSA staining at the equatorial segment were identified as those with acrosome reactions.

### Statistical Analysis

Data were tested for homogeneity of variance by Bartlett’s test and for normal distribution by the Shapiro-Wilk test. One-way ANOVAs were performed with Tukey’s multiple comparisons tests or Dunnett’s multiple comparisons tests as appropriate. Data from the CASA system were compared by the Friedman test and Dunn’s multiple comparison test. All statistical analyses were performed using GraphPad Prism 6 (La Jolla, CA, USA). The results are expressed as mean ± SEM. Values were determined to be significant when * p < 0.05, ** p < 0.01, and *** p < 0.001, **** p < 0.0001, indicating a significant difference between the groups and control (p < 0.05).

## Results

### FGF21 Concentrations in Seminal Fluid

**Figure 1:**
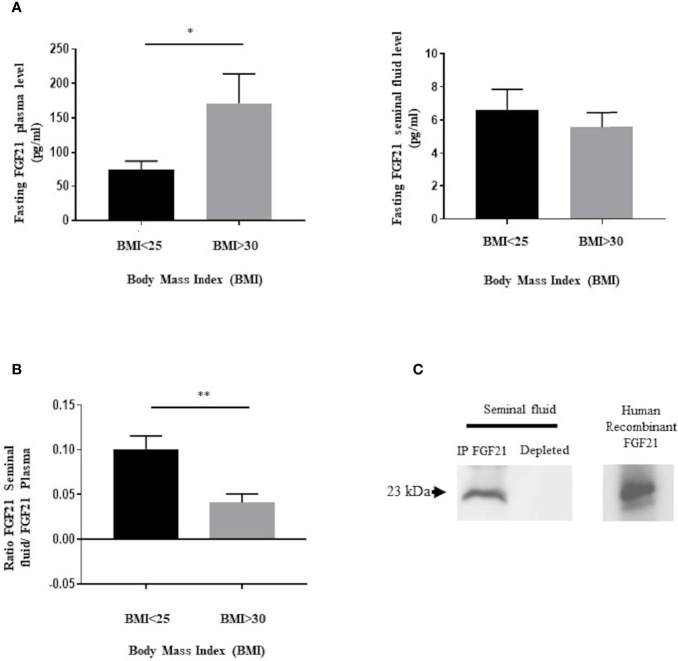
Presence of FGF21 in human plasma and seminal fluid. **(A)** FGF21 was determined, from two groups of fasting patients, in plasma (n = 10/BMI group) and in seminal fluid (n = 12/BMI group), in function of their body mass index (BMI). Control corresponding to a BMI ≤ 25 kg/m^2^, and obesity corresponding to a BMI ≥ 30 kg/m^2^. **(B)** Ratio of fasting FGF21 level between seminal fluid and plasma in the two groups of patients. **(C)** Immunoblot of a pool of human seminal fluid protein extracts (400 μg protein per sample) after immunoprecipitation with the FGF21 polyclonal antibody. Human recombinant FGF21 was loaded as the control to confirm the molecular weight of the immunoprecipitated FGF21 protein. *p < 0.05, **p < 0.01.

Firstly, we have investigated the presence of FGF21 in human seminal fluid by an ELISA and immunoprecipitation assay. We have analyzed the FGF21 levels in fasting conditions in blood and seminal fluid from two groups of patients using the BMI, a normal BMI in the range 18.5–25 kg/m^2^ and with obese patients with a BMI ≥ 30 kg/m^2^ (**Figure 1** and **Table 1**). FGF21 plasma levels were 2.4-fold higher in obese patients in comparison to control patients (**Figure 1A**). However, a similar FGF21 level was measured in the seminal fluid of both groups (**Figure 1A**). We observed that the FGF21 concentration was nearly 12 to 28 times lower in the seminal fluid as compared to that in the plasma, and the FGF21 seminal fluid/FGF21 plasma ratio was lower in obese patients (**Figure 1B**). Due to the lower levels in seminal fluid, we had confirmed the presence of FGF21 in seminal fluid after immunoprecipitation and its absence in the depleted protein extract (**Figure 1C**). No significant relationship between plasma or seminal FGF21 levels and semen parameters (semen volume, sperm concentration, motility, or sperm abnormality) was observed (**Table 1** and **Supplementary Data Sheets 1**, **2**).

**Figure 2:**
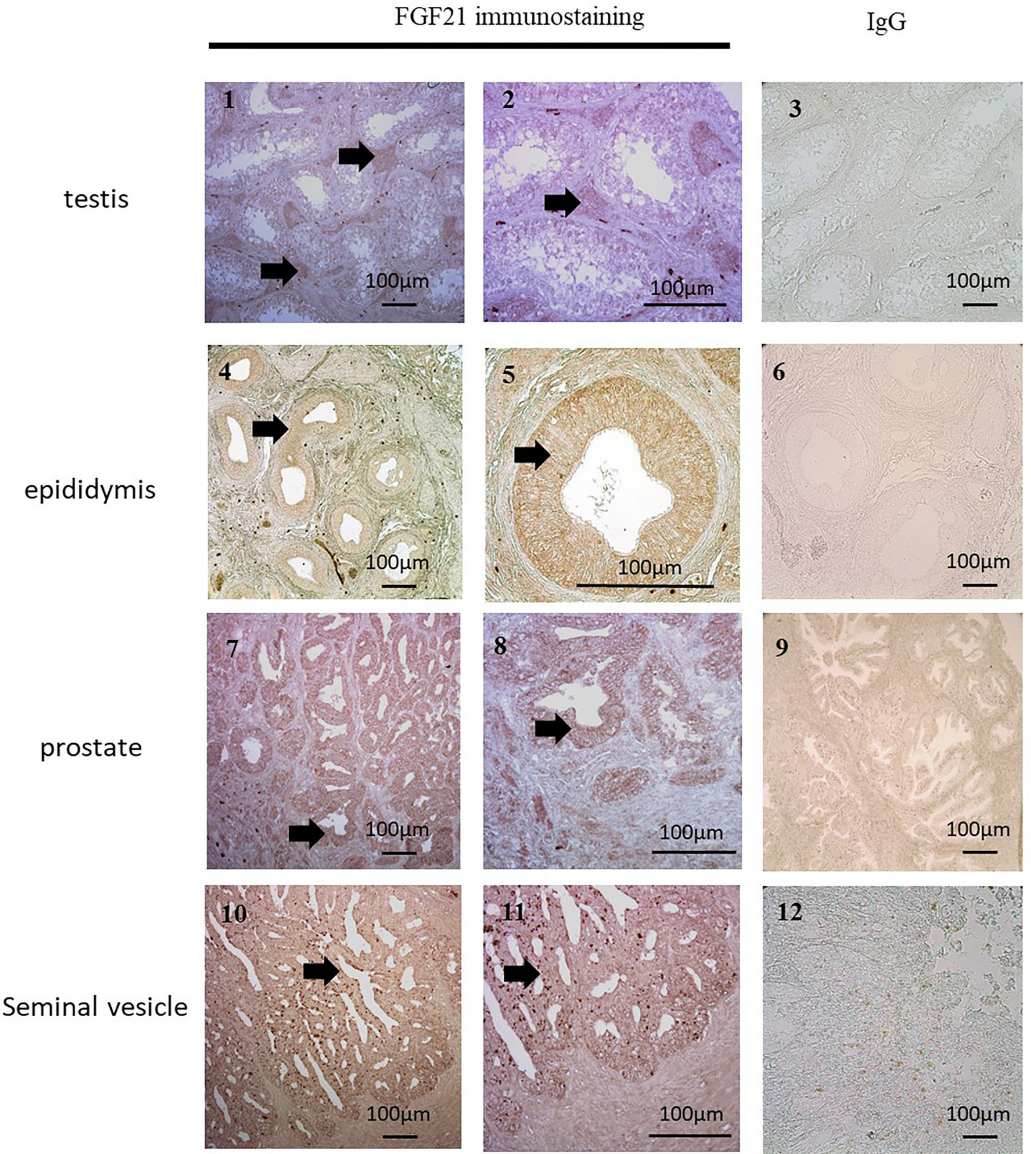
Localization of FGF21 in reproductive organs. FGF21 was localized by immunohistochemical staining in human testis (1 and 2), epididymis (4 and 5), seminal vesicles (7 and 8) and prostate (10 and 11). Negative controls (3, 6, 9, and 12) were sections incubated with IgG. We observed FGF21 expression in Leydig cells and the epithelia of the epididymis, prostate and seminal vesicles (see arrow), which contained all secretory cells.

**Figure 3:**
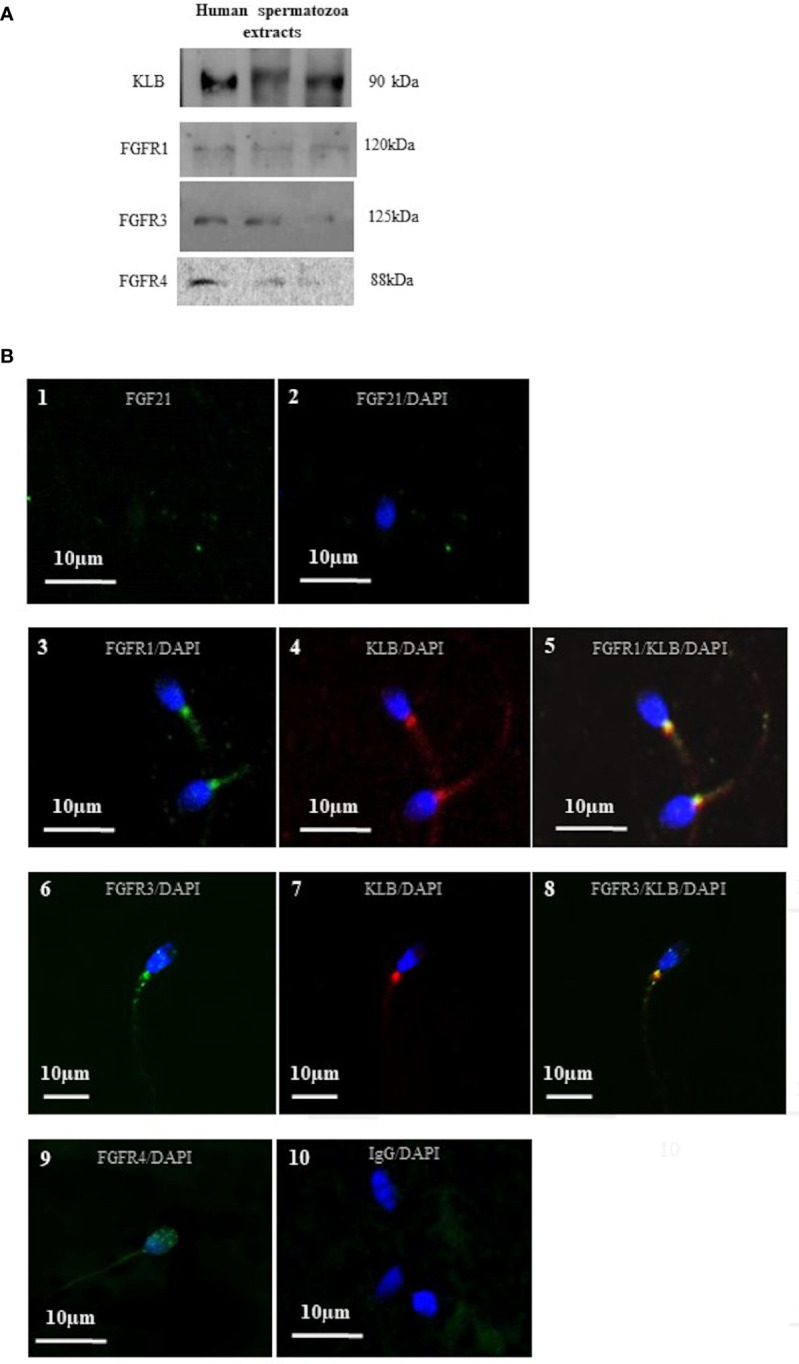
Localization of FGF21 receptors on human spermatozoa. **(A)** Detection of sperm cofactor KLB and the FGFR1, FGFR3, and FGFR4 using Western immunoblotting. Red ponceau was used to check protein deposition on the membrane. Each lane represents extracts from different donors. Receptors and cofactor were tested on different gels. **(B)** FGF21 (1 and 2), FGFR1, FGFR3, FGFR4 (3, 6, and 9), and KLB (4 and 7) localization in human spermatozoa were analyzed by confocal microscopy after immunofluorescence. FGFR1, FGFR3 and FGFR4 were stained by Alexa Fluor 488 goat anti-rabbit IgG (green) and KLB (4 and 7) by Alexa Fluor 633 rabbit anti-mouse IgG (red). Merged picture of FGFR1 and FGFR3 with KLB showing the colocalization in the mid-piece of spermatozoa (5 and 8). Negative control was incubated with IgG (10). DNA was counterstained with DAPI.

Because the majority of seminal fluid proteins are produced by the epididymis, seminal vesicles and prostate, immunochemistry against FGF21 has been performed on the male reproductive tract (**Figure 2**). The expression of FGF21 was reported in Leydig cells in the testis, in the epithelium of the epididymis, and in the seminal vesicles, with weaker staining in the epithelium of the prostate gland (**Figure 2**). However, no staining was observed in human spermatozoa (**Figure 3B**.1).

### FGF21 Receptors and Cofactor Are Localized in Spermatozoa

As reported in the literature, FGF21 signaling is transduced by activating the β-klotho-FGFR1c/FGFR3 complex, which stimulates the components of the MAPK and PI3K pathways, and the calcium-dependent protein. In our condition, we have confirmed, by Western blot, the presence of FGFR1, FGFR3, FGFR4 and KLB proteins in human spermatozoa (**Figure 3A**). In order to localize the FGFR complex, a confocal microscopic analysis was performed and demonstrated the colocalization of the two FGF21 receptors (FGFR1 and FGFR3) with the cofactor KLB (**Figure 3B**; 5, 8) in the middle piece of the spermatozoa, behind the head and weakly in the tail. Weak staining of the FGFR4 receptor was reported in the head and in the neck of the spermatozoa (**Figure 3B**; 9).

**Figure 4:**
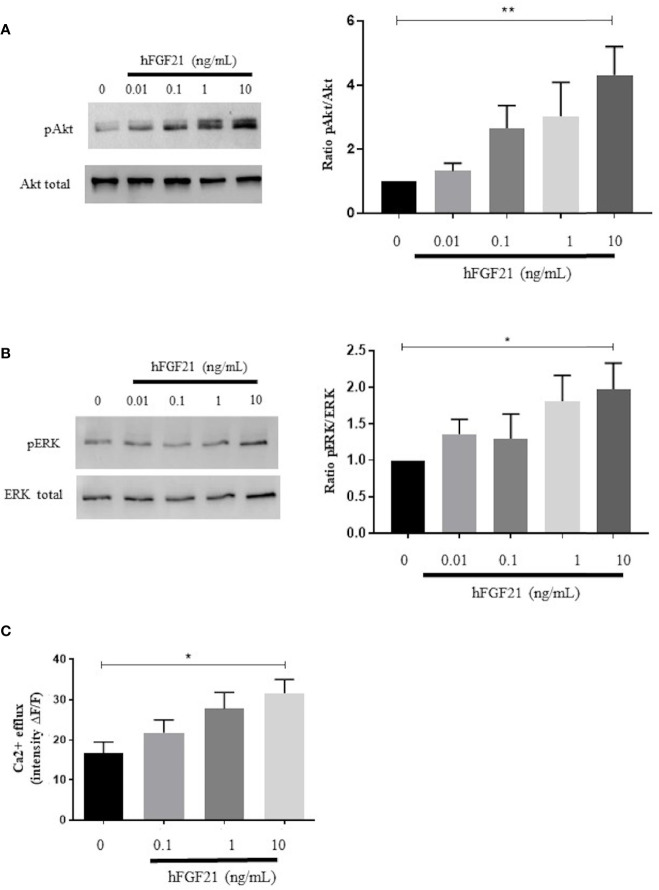
Signaling pathways activated by the exposure of human spermatozoa to FGF21. **(A, B)** Western blot and analysis of phosphorylated (ser473) Akt and phosphorylated (Thr202/Tyr204) ERK in human spermatozoa exposed to recombinant FGF21 (0.01-10 ng/mL), as described in Materials and Methods section. Results are representative of at least four independent experiments. **(C)** Intracellular calcium responsiveness of sperm to recombinant FGF21 stimulation determined by fluorescence intensity relative to baseline (ΔF/F) at 30 min. *p < 0.05, **p < 0.01.

After 15 min of stimulation of fresh human spermatozoa with increasing concentrations of recombinant human FGF21 (0.01 ng/mL-10 ng/mL), we observed a dose-dependent increase in the phosphorylation of both Akt and ERK, which was significant at the 10 ng/mL FGF21 concentration (in comparison to control, phospho-Akt had a 4.3-fold increase and phospho-ERK had a 2-fold increase) (**Figures 4A, B**). We observed that, at the low dose of 0.1 ng/mL, phospho-Akt increased 2.6-fold, but this increase was statistically insignificant. In addition, we observed that 15 min after exposure to recombinant human FGF21, the intracellular flux of Ca^2+^ was increased in a dose-dependent manner and significantly at the 10 ng/mL concentration of FGF21 (**Figure 4C**).

### FGF21 Increased Sperm Motility

To determine the effect of FGF21 on spermatozoa, we incubated fresh human spermatozoa with recombinant FGF21 for 30 min. The investigation of time effect was performed on spermatozoa with progressive motility, as presented in **Supplementary Data Sheet 3A**, showing that 30 min is the optimal time. No consequence on the viability of sperm after FGF21 stimulation was measured by SYBR-14 or propidium iodide staining (**Supplementary Data Sheet 3B**).

**Figure 5:**
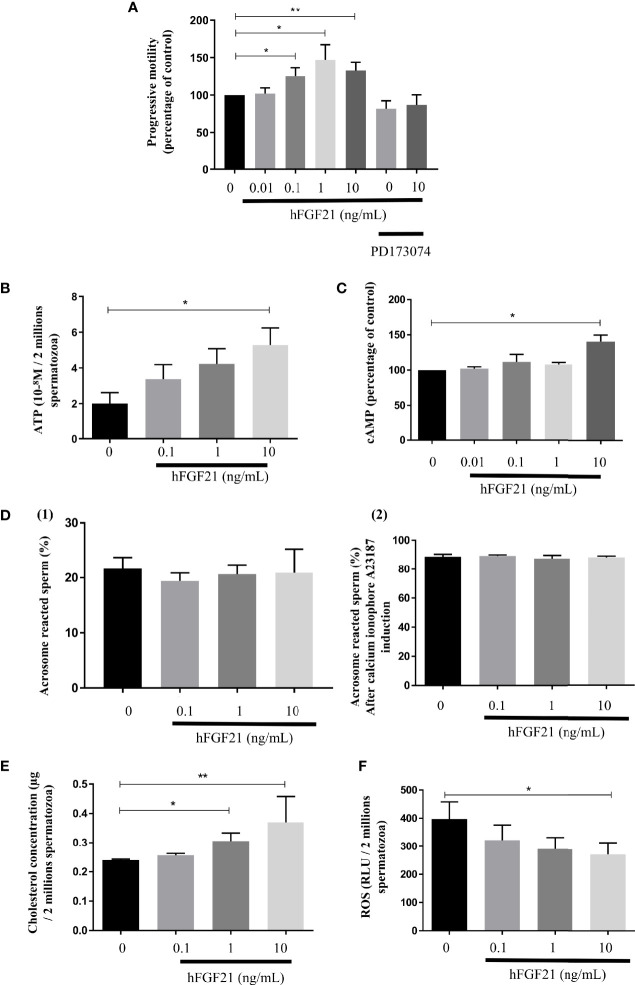
FGF21 treatments increase spermatozoa motility. **(A)** Sperm progressive motility analyzed by computer-assisted sperm analysis (CASA) after 30 min of stimulation with recombinant FGF21, was presented in percentage of control. A preincubation with the FGFR inhibitor PD173074 was also used in presence of absence of a 30 min of stimulation with recombinant 10 ng/mL FGF21, (n = 8 patients). **(B, C)** Concentration of ATP in sperm stimulated for 30 min with recombinant FGF21 (10^-8^M per 2.10^6^ cells) and cAMP production in percentage of control, (n = 7 patients). **(D)** Percentage of acrosome-reacted sperm with (2) or without (1) calcium ionophore A23187 was quantified after PSA staining (percentage of PSA negative cells). Acrosome reaction of spermatozoa after 30 min of stimulation with recombinant FGF21 (n = 5 patients). **(E)** Total cholesterol concentration quantified in human spermatozoa after 30 min of stimulation with recombinant FGF21 (µg per 2.10^6^ cells, n = 7 patients). **(F)** ROS levels in human spermatozoa incubated with increasing concentrations of recombinant FGF21 for 30 min (relative luminescence units per 2.10^6^ cells, n = 7 patients). *p < 0.05, **p < 0.01.

We have analyzed the effect of FGF21 on the motility of sperm motility after exposing human spermatozoa to FGF21 for 30 min at 37°C. Compared to the control condition, FGF21 was able to significantly increase progressive motility at the 0.1 ng/mL and higher concentrations of FGF21 (**Figure 5A**). Preincubation with the FGFR inhibitor PD173074 was able to eliminate the stimulatory effect induced by 10 ng/mL FGF21 (**Figure 5A**). Moreover, the average velocity and curvilinear line velocity of the spermatozoa determined by VCL and VAP were also improved by about 20% to 27% (0.1-10 ng/mL FGF21) and were returned to control values if a preincubation with PD173074 was performed (**Table 2**). Because motility is highly associated with mitochondria activity, the ATP level and mitochondrial membrane potential were analyzed. Despite spermatozoa still having a high mitochondrial membrane potential (control: 45.71% ± 1.89; FGF21: 0.1 ng/mL: 46.33% ± 2.12; FGF21: 1 ng/mL: 47.19% ± 2.19; FGF21: 10 ng/mL: 46.72% ± 2.15) (**Supplementary Data Sheet 4**), a significant increase of both the ATP and cAMP levels in the spermatozoa was measured at the 10 ng/mL FGF21 concentration as compared to those in the control (**Figures 5B, C**).

Furthermore, we did not observe any consequence of FGF21 exposure to the already high percentage of acrosome-reacted sperm induced with by calcium ionophore [**Figure 5D**-(2)] or without [**Figure 5D**-(1)]. But, FGF21 improved oxidative stress by reducing the levels of reactive oxygen species (ROS) in a dose-dependent manner in human sperm (**Figure 5F**). Moreover, the stimulation of sperm by FGF21 induced a significant increase in cholesterol levels (**Figure 5E**).

## Discussion

**Figure 6:**
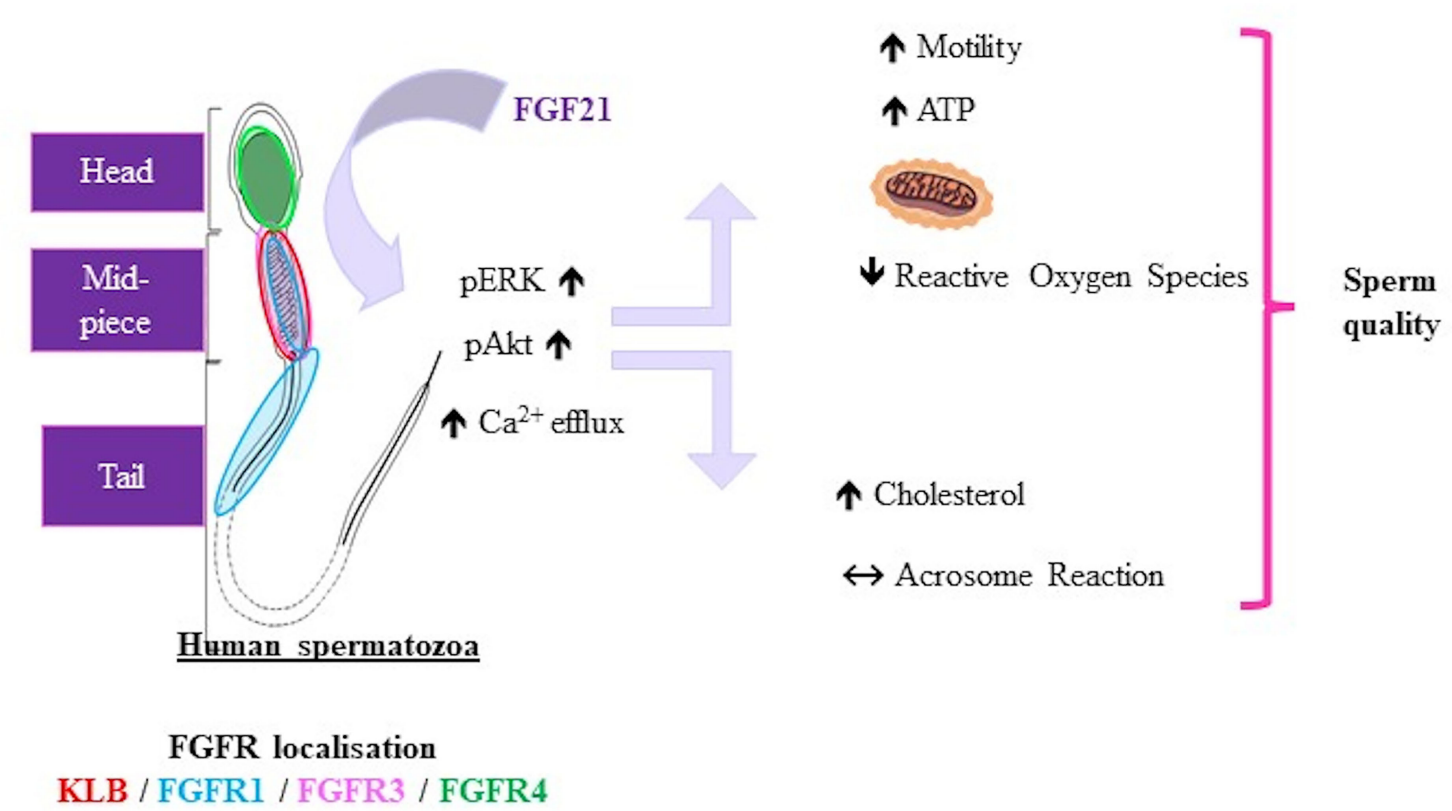
Representative schema of FGF21 effects on human spermatozoa. Representative schema of the localization of FGFRs and KLB in spermatozoa and effects of FGF21 on human sperm. FGF21 induced phosphorylation of the Akt and ERK pathways and calcium levels, then increased the sperm motility associated with ATP content in a dose-dependent manner, and reduced oxidative stress.

We demonstrated the presence of FGF21 in human seminal fluid and argued that seminal FGF21 could be produced by the different tissues of the male reproductive tract. Our immunohistochemical studies on human sperm revealed that FGF21 can transduce signaling by activating the β-klotho-FGFR1c or FGFR3 complex in the mid-piece of spermatozoa. The *in vitro* sperm stimulation by FGF21 leads to increased mobility by boosting the production of ATP in spermatozoa, and reduced oxidative stress. FGF21 activated in a dose-dependent manner the Akt and ERK phosphorylation and modification of the calcium efflux (**Figure 6**).

As in the literature, significant variations in plasma concentrations of FGF21 were measured: between 50 and 5 000 pg/mL in humans (9) and between 100 and 1 000 pg/mL in mice (22). We showed the presence of FGF21 in seminal fluid with concentrations 20-fold lower than that in the plasma. Interestingly, we observed that the levels of FGF21 in seminal fluid are independent of the BMI, semen volume, sperm concentration or sperm abnormalities. Changes in FGF21 plasma levels due to BMI were not recovered in seminal fluid. This raises two hypotheses regarding its origin. Firstly, FGF21 crosses (passively or actively) the testicular barrier from blood or secondly, seminal FGF21 is produced by local cells. Because the seminal vesicles secrete up to 75% (23) of the total volume of seminal fluid and are also completed by the prostate gland, we performed immunohistochemical studies of FGF21 in these human glands. The strong staining of FGF21 in human epididymis and seminal vesicle samples is coherent with the physiological role of these tissues. In hepatocytes, FGF21 is known to be regulated by starvation and PPARα activity. Interestingly, PPARα has already been described to be expressed specifically in epithelial cells of the prostate, which, in our study, also expressed the FGF21 protein (24, 25). To elucidate the origin of FGF21 in seminal fluid, the use of transgenic mouse lines with conditional knockouts could better determine if FGF21 is produced locally or not.

FGF21 preferentially activates FGFR1 and FGFR3, with the recruitment of the specific β-klotho cofactor (8). In our conditions, in human spermatozoa, we colocalized the FGFR1, FGFR3, and β-klotho in the mid-piece of the spermatozoa. Then, the machinery for signal transmission of FGF21 is in place in the part of spermatozoa that contain the mitochondria. These results are similar with previous immunocytochemical studies that allowed the localization of FGFR1, 2, 3 and 4 in ejaculated human sperm (17). In adipocytes, activation of receptors by FGF21 leads to phosphorylation of MAPK (ERK) and Akt (26, 27). As in adipocytes, we report, in human semen, an increase in phosphorylated (ser473) Akt and phospho-(Thr202/Tyr204) ERK. Some components of these pathways, such as ERK, PI3K and Akt, have been described to play an essential role in the maintenance of sperm function in mammalian sperm (28, 29). It has been shown that inhibition of Akt decreases sperm motility in mice, and activation of Akt stimulates sperm motility in humans (17, 30, 31). In somatic cells, the FGF/FGFR1 system has been shown to facilitate cell motility and migration by activation of the PI3K and ERK pathways (15, 32). From these data, it is not surprising to observe an enhancement of ATP levels in spermatozoa, as well as an increasing percentage of motile sperm. Sperm requires exceptionally high amounts of ATP when compared to somatic cells (33). Interestingly, an autocrine FGF (FGF2) has been reported to be present directly in human spermatozoa and 10 ng/mL recombinant FGF2 is able to enhance the motility of sperm (34). We notice that FGF2 needs a different receptor complex for FGF21, using the HS cofactor.

In the case of metabolic syndrome, these observations raise questions about the expression and the role of the endocrine FGF21 factor in sperm. Currently, a protective role for FGF21 is advanced, as well as multiple positive actions, and FGF21 could lead to the activation antioxidant pathways in targeted cells (35). In our study, we investigated the levels of ROS in human semen and describe a decrease in the ROS level 30 min after sperm stimulation by FGF21, suggesting a putative role in the observed improvement in motility. Indeed, the high susceptibility of sperm cells to ROS results from the composition of its membranes, which is rich in polyunsaturated fatty acids (PUFA) and thus highly susceptible to attack by ROS (36, 37). The low ROS levels after FGF21 treatment suggests a possibility to improve the quality and/or motility of human spermatozoa (38).

Events that are associated with capacitation include elevation of intracellular Ca^2+^, higher levels of intracellular cAMP, and cholesterol efflux from the membranes of sperm that increases membrane fluidity (39). In sperm, Ca^2+^ plays a central role in the events preceding fertilization, such as motility, chemotaxis, and the acrosome reaction. In our conditions, we observed a FGF21 dose-dependent increase in Ca^2+^ efflux in spermatozoa; however, because the acrosome reaction was already elevated, no change in the acrosome reaction was noted. In the same way, the membranes of sperm and cholesterol efflux contribute to mechanisms that control sperm capacitation (40, 41). We observed a significant increase in cholesterol levels after FGF21 stimulation. In somatic cells, FGF21 promotes the efflux of cholesterol (42). If we transpose this knowledge to spermatozoa, we can hypothesize that FGF21 promotes cholesterol efflux, which can occur to the capacitation process.

The hepatokine FGF21 can be associated with other similar metabolic signals called adipokines, which have recently been shown to be involved in male fertility. Over the last decade, several adipokines have been detected in seminal fluid (43, 44) and have been shown to have a role in sperm functions, such as leptin (which is found to enhance sperm capacitation). Similarly to FGF21, differences in the concentrations of adipokines between seminal fluid and plasma have been reported with lower or enhanced concentrations in seminal fluid (45–47). It would be interesting to see if the concentrations of FGF21 in seminal fluid are associated with metabolic markers in blood and could be used as a biomarker related to fertility. Likewise, a better identification of the origin of FGF21 (between local secretion and peripheral) would make it possible to not only use the circulating rates as a predictor of the quality of male fertility but also to know whether the use of an FGF21 agonist would impact the spermatozoon.

In conclusion, we propose the endocrine factor FGF21 as a novel regulator of male reproductive function with direct actions on germ cells. FGF21 is able to improve sperm motility, oxidative stress, and markers of capacitation. FGF21 is then involved in the crosstalk between human metabolism and spermatogenesis.

## Data Availability Statement

The original contributions presented in the study are included in the article/**Supplementary Material**. Further inquiries can be directed to the corresponding author.

## Ethics Statement

Patients were enrolled into the Assisted Reproductive Centers (Tours, FERTIPROTECT protocol) for couple infertility exploration during a medical consultation. Forty participants gave full-informed written consent to participate in the study, and ethical approval was obtained from the Ethics Committee of the Vinci Clinic and CHRU Bretonneau. The patients/participants provided their written informed consent to participate in this study.

## Author Contributions

GB and AE performed the study. CC and CR were technical support. FG, J-SB, CV, IP, EC-S are in charge of patients included in the study. GF and DDR provided human histological sections. JD participated in the review. PF and PHD designed the study and performed experiments. All authors contributed to the article and approved the submitted version.

## Funding

GB was supported by the Region Centre Val de Loire and INRAE PHASE. This work was financially supported by the national program « INDICA » funded by the APR Region Centre Val de Loire.

## Conflict of Interest

The authors declare that the research was conducted in the absence of any commercial or financial relationships that could be construed as a potential conflict of interest.

## Publisher’s Note

All claims expressed in this article are solely those of the authors and do not necessarily represent those of their affiliated organizations, or those of the publisher, the editors and the reviewers. Any product that may be evaluated in this article, or claim that may be made by its manufacturer, is not guaranteed or endorsed by the publisher.

## Supplementary Material

Supplementary Data Sheet 1 Biological and semen parameters of samples from all patients (n=40). Data are expressed as mean ± SEM (range).

Supplementary Data Sheet 2 Correlations between FGF21 levels (plasma or seminal fluid) and sperm parameters (n=20).

Supplementary Data Sheet 3
**(A)** Investigation of time effect on spermatozoa progressive motility. The optimal time to observe a change in sperm motility, in FGF21 (1ng/ml) condition was after 30 min of stimulation. All results are expressed as mean ± SEM (n = 4). *p < 0.05, **p < 0.01, Mann-Whitney Test. **(B)** Effect of FGF21 on semen viability was determined by a double-fluorescent labeling technique (SYBR-14 and propidium iodide). The PI negative and SYBR-14 positive population showing green fluorescence was considered alive. Samples were analyzed using flow cytometry (MoFlo AstriosEQ, USA).

Supplementary Data Sheet 4 Mitochondrial activity was determined using a 200 nM mitotracker (Orange CM-H2TMRos, Invitrogen, Fisher Scientific, France). Samples were analyzed using flow cytometry (MoFlo AstriosEQ, USA). Twenty thousand events were collected per sample. Only sperm emitting red fluorescence were classified with a high mitochondrial membrane potential (HMMP), which is associated with high mitochondrial activity. The ‘R2’ window is Mitotracker negative cells and the ‘R1’ window is Mitotracker positive cells.
