# Supplementary figures and images for: The Hepatokine FGF21 Increases the Human Spermatozoa Motility
Source: Front Endocrinol (Lausanne). 2022 Feb 24;13:775650. doi: 10.3389/fendo.2022.775650 (PMC8908098; doi:10.3389/fendo.2022.775650)

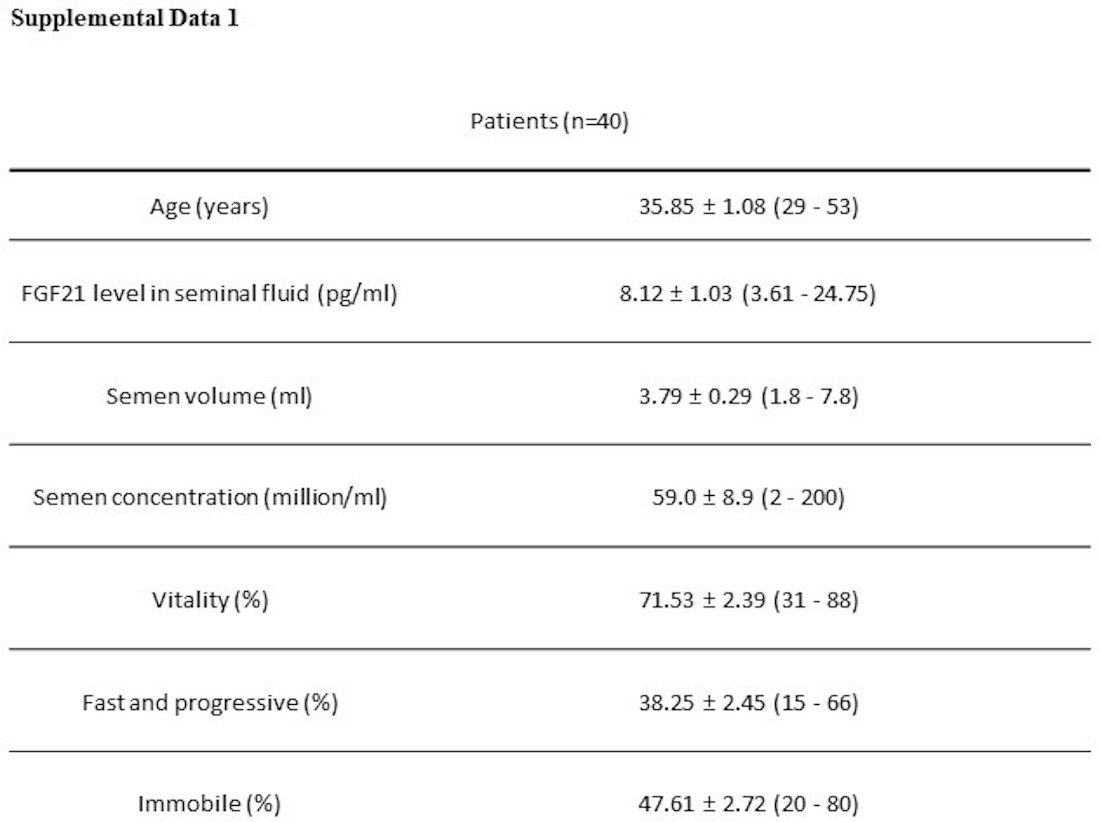

Supplement: Supplementary Data Sheet 1 — Biological and semen parameters of samples from all patients (n=40). Data are expressed as mean ± SEM (range). [file Image_1.jpeg]

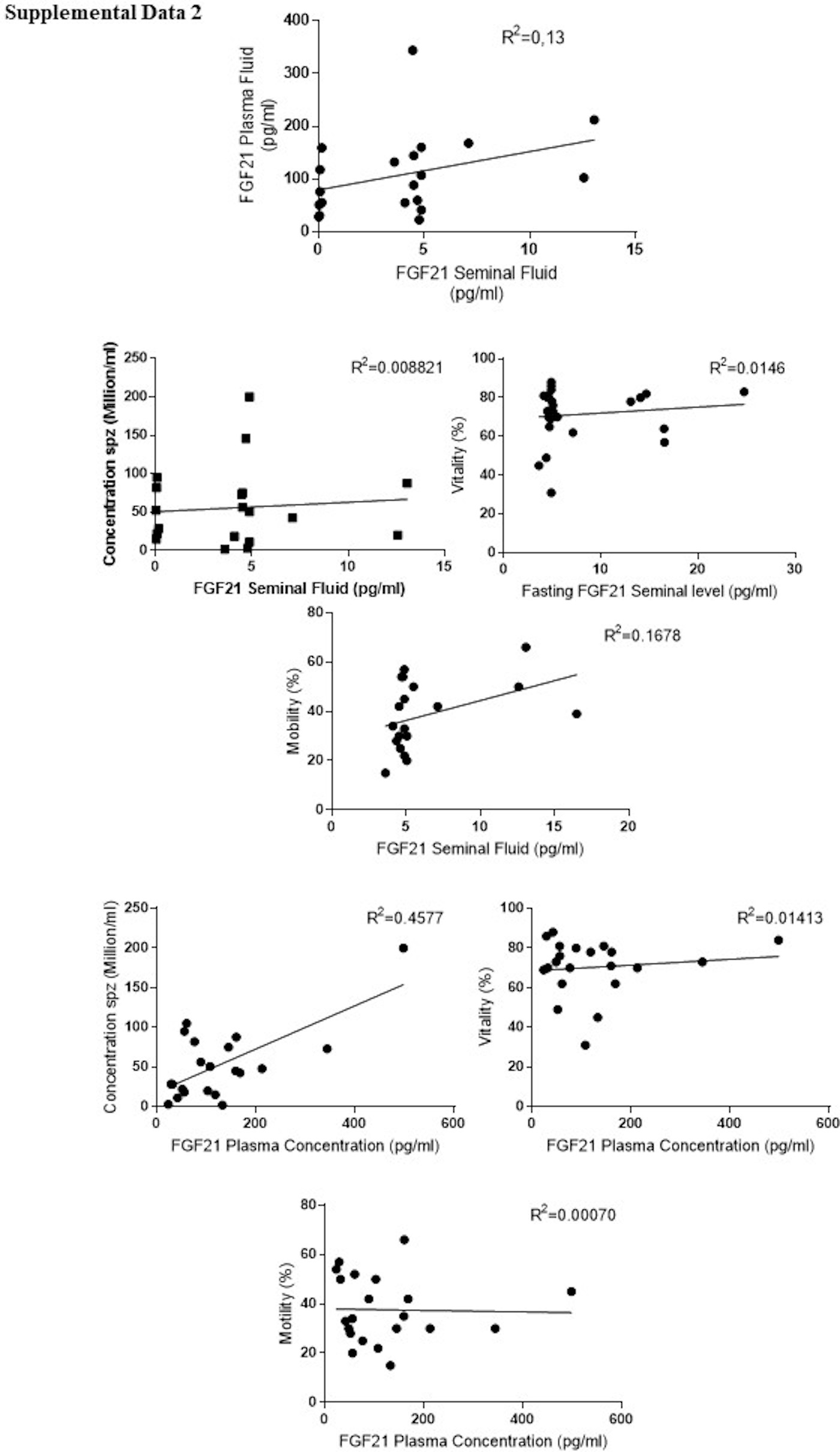

Supplement: Supplementary Data Sheet 2 — Correlations between FGF21 levels (plasma or seminal fluid) and sperm parameters (n=20). [file Image_2.jpeg]

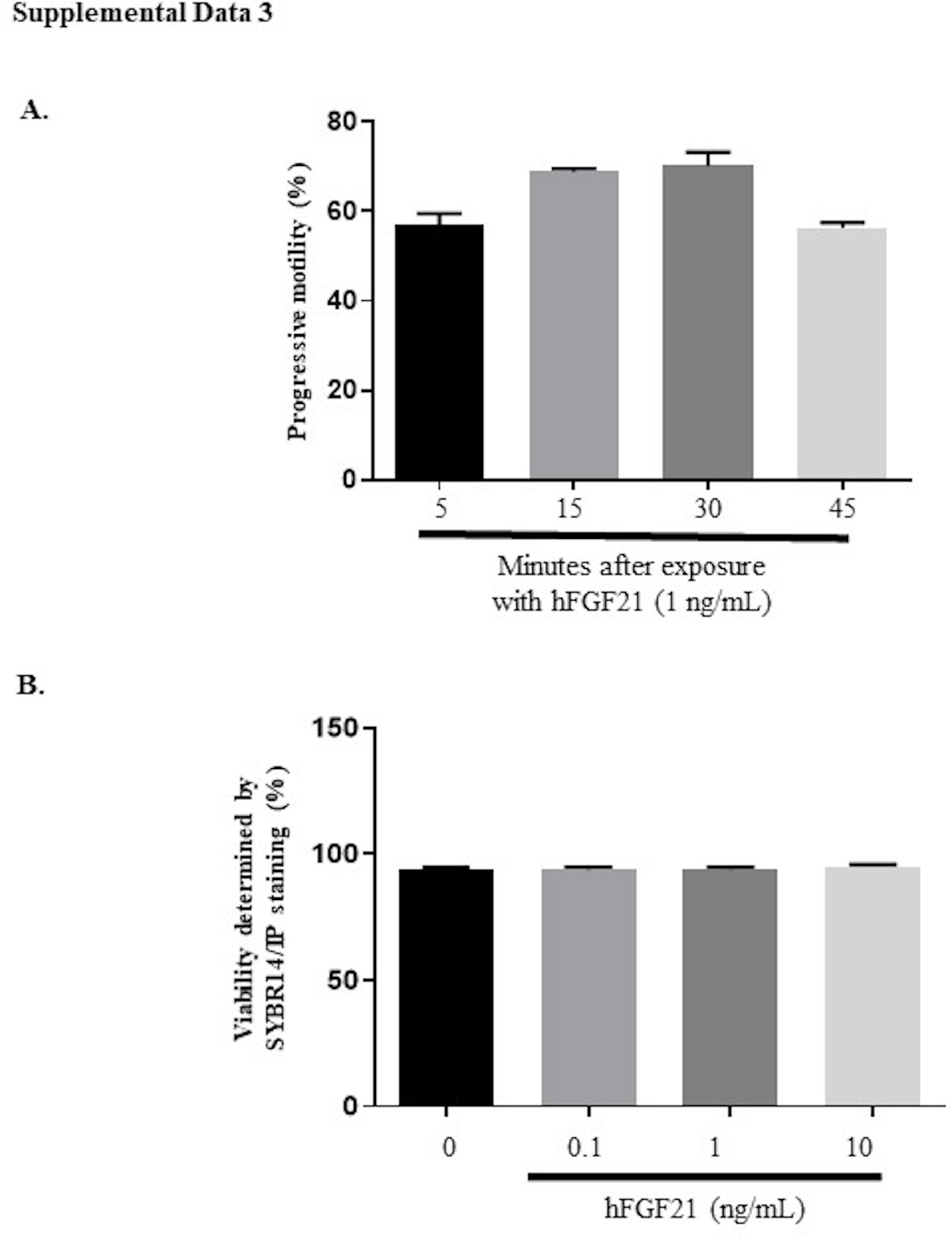

Supplement: Supplementary Data Sheet 3 — (A) Investigation of time effect on spermatozoa progressive motility. The optimal time to observe a change in sperm motility, in FGF21 (1ng/ml) condition was after 30 min of stimulation. All results are expressed as mean ± SEM (n = 4). *p < 0.05, **p < 0.01, Mann-Whitney Test. (B) Effect of FGF21 on semen viability was determined by a double-fluorescent labeling technique (SYBR-14 and propidium iodide). The PI negative and SYBR-14 positive population showing green fluorescence was considered alive. Samples were analyzed using flow cytometry (MoFlo AstriosEQ, USA). [file Image_3.jpeg]

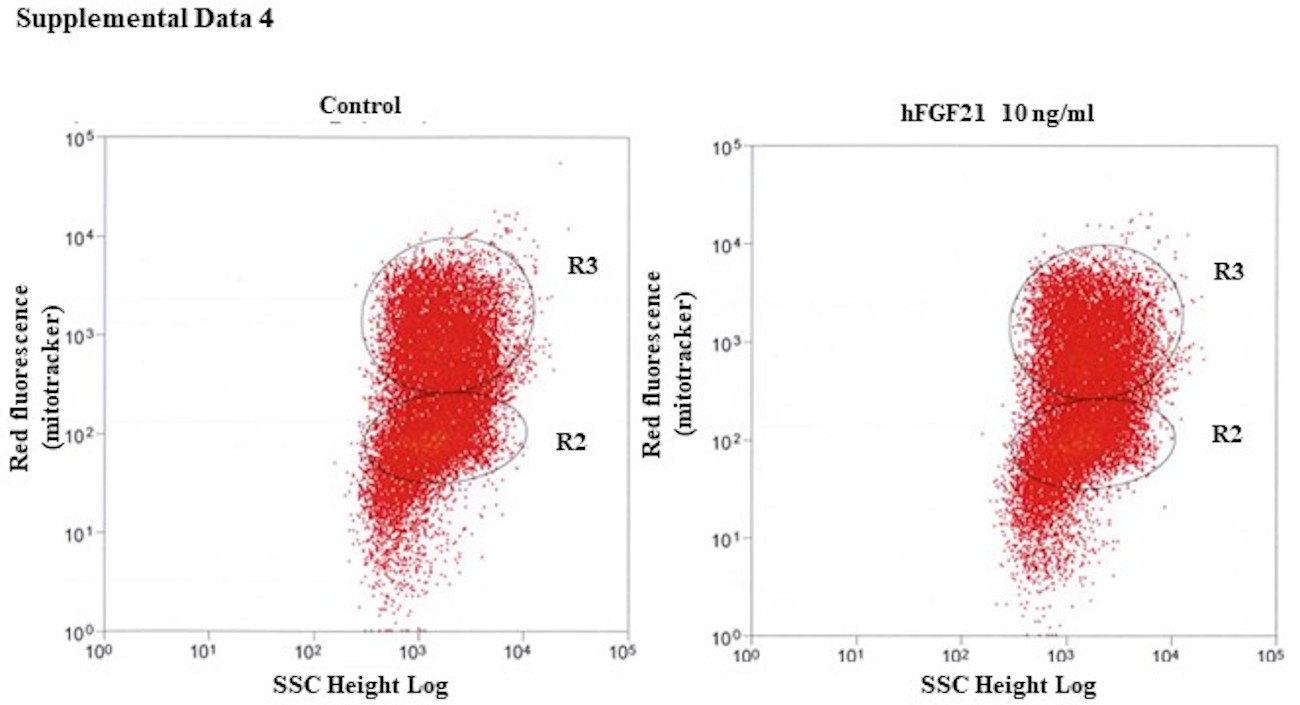

Supplement: Supplementary Data Sheet 4 — Mitochondrial activity was determined using a 200 nM mitotracker (Orange CM-H2TMRos, Invitrogen, Fisher Scientific, France). Samples were analyzed using flow cytometry (MoFlo AstriosEQ, USA). Twenty thousand events were collected per sample. Only sperm emitting red fluorescence were classified with a high mitochondrial membrane potential (HMMP), which is associated with high mitochondrial activity. The ‘R2’ window is Mitotracker negative cells and the ‘R1’ window is Mitotracker positive cells. [file Image_4.jpeg]
